# Notes of M. Bernard’s Lectures on the Blood

**Published:** 1854-12

**Authors:** 


ART. IX.—*Notes of M. Bernard's Lectures on the Blood; with an Appendix.* By WALTER F. ATLEE, M.D. Philadelphia: Lippincott, Grambo & Co. 1854.

We have delayed our notice of this little book, in hopes that time might be afforded for a careful perusal of its contents. It is not so large as to make it tedious, but the days have gone by and only a portion of its contents have been digested.

Enough has already been read to give us some idea of its contents. Dr. Atlee has given us his notes of Bernard's Lectures, and consequently we have a very systematic little treatise, condensed to a fault, without any waste matter, each sentence being a natural sequence to its predecessor, and depending upon it for its full appreciation. These are not bad qualities, and to the student of physiological chemistry the book presents as much material as might easily be inflated to a ponderous octavo.

All our readers know who M. Bernard is, and we have only to announce that this is on sale to secure for it purchasers. There is no living physiologist who is accomplishing so much for his science as Bernard. He is still young, and has rapidly risen to a most enviable position, due alone to his worth and labors. He is no theorizer, but he is a most careful and accurate experimenter; and from these experiments he has educed doctrines which are already exerting a vast influence upon therapeutics and pathology. Everything that Bernard does seems to have attached to it an immediate and practical value.

His doctrine of digestion, supported as it is by the most logical experiment, is alone enough to immortalize his name, and in perusing it we have a satisfactory feeling that the end is reached, that no double set of conclusions are to be drawn from his labors.

The appendix is made up of notes referring to the body of the work, and drawn entirely from the researches of M. Robin, the leading microscopist of Paris at the present day. M. Robin is as prominent in this department as Bernard in vital chemistry; so that we have, here, in a little duodecimo, the leading results of the labors of two men, the foremost in their closely allied departments, the whole making the latest treatise on the anatomy of the blood within reach of the reading public. Dr. Atlee has done an essential favor to the profession by the publication of his notes, and we wish for them a large circulation.

For sale by MILLER, ORTON, & MULLIGAN. H.

